# Late Exercise Preconditioning Promotes Autophagy against Exhaustive Exercise-Induced Myocardial Injury through the Activation of the AMPK-mTOR-ULK1 Pathway

**DOI:** 10.1155/2019/5697380

**Published:** 2019-11-24

**Authors:** Hong-Tao Liu, Shan-Shan Pan

**Affiliations:** School of Kinesiology, Shanghai University of Sport, Shanghai, China

## Abstract

Accumulating evidence shows that the AMPK-mTOR pathway modulates autophagy via coordinated phosphorylation of ULK1. The aim of the present study was to investigate the relationship between AMPK, mTOR, and ULK1 during late exercise preconditioning (LEP), and to explore whether LEP-induced myocardial protection is related to the autophagy. The exercise preconditioning (EP) protocol was as follows: rats were instructed to for run four repeated in duration of 10 minutes (including 10 minutes rest between each period) on a treadmill. Exhaustive exercise (EE) after LEP pretreatment and administration of wortmannin (an autophagy inhibitor that suppresses Class III PI3K-kinase (PI3KC3) activity) were added to test the protective effect. Cardiac troponin I (cTnI), and transmission electron microscopy (TEM), along with hematoxylin-basic fuchsin-picric acid (HBFP) staining, were used to evaluate the myocardial ischemic-hypoxic injury and protection. Western blot was used to analyze the relationship of autophagy-associated proteins. Exhaustive exercise caused severe myocardial ischemic-hypoxic injury, which led to an increase in cTnI levels, changes of ischemia–hypoxia, and cells ultrastructure. Compared with the EE group, LEP significantly suppressed exhaustive exercise-induced myocardial injury. However, wortmannin attenuated LEP-induced myocardial protection by inhibiting autophagy. Compared with the C group, AMPK was increased in the LEP, EE, and LEP+EE groups, but phosphorylation of AMPK at Thr172 was not significantly changed. Exercise did not have any effect on mTOR expression. Compared with the C group, ULK1 was increased and the ULK1^ser757^/ULK1 ratio was decreased in the LEP and LEP+EE groups. ULK1 was not significantly affected in the EE group, however, phosphorylation of ULK1 at Ser757 was remarkably decreased. To sum up, our results suggested that LEP promoted autophagy through the activation of AMPK-mTOR-ULK1 pathway, and that activated autophagy was partially involved in myocardial protection against EE-induced myocardial ischemic-hypoxic injury.

## 1. Introduction

Acute exercise-induced cardiac preconditioning has confer immediate cardioprotection against ischemic events [1]. Repeated high-intensity interval exercise may cause repeated relative or absolute myocardial ischemia–hypoxia, which in turn enhances cardiomyocytes tolerance [2–4]. This form of exercise-induced intrinsic myocardial protection is termed exercise preconditioning (EP), which exerts myocardial protective effects on subsequent permanent myocardial ischemic-hypoxic injury. The intrinsic myocardial protection initiated by EP is similar to ischemic preconditioning (IPC), and may cause reduction of myocardial stunning [5], and the reduction in infarct size [6, 7]. Similar to IPC, EP has two protection phases: early exercise preconditioning (EEP) and late exercise preconditioning (LEP). After EP, EEP occurs immediately and sustains 2 ~ 3 hours, and LEP emerges 24 hours later and can last several days [1]. Previous studies have discovered a number of factors that are associated with late myocardial protection of EP, including PKC family proteins, mitochondrial KATP channels, and mitophagy [2, 8, 9]. Nevertheless, the underlying mechanisms of EP-induced myocardial protection are still not fully understood.

Autophagy, a lysosome dependent degradation process, contributes to maintenance of energy balance and organelle renewal in the cells [10]. In mammals, autophagy may be activated by fasting, ischemia/reperfusion (I/R), or physical exercise [11–13]. A previous study has reported that IPC-induced autophagy can exert cardioprotective effects by removing damaged intercellular organelles [14].

ULK1 complex is a required macromolecular complex for activation of autophagy [15, 16]. ULK1 activation is negatively regulated by mTOR and positively regulated by AMPK [17]. During normal conditions, autophagy inhibitor mTOR phosphorylates ULK1 at Ser757 to block the interaction of AMPK-ULK1, thereby inhibiting autophagy [18]. Upon autophagy induction, ULK1 is dephosphorylated at Ser757, and then separated from mTOR and activated [19]. AMPK, a second level of ULK1 regulation, is activated at the time of autophagy induction. As an energy-sensitive enzyme, AMPK is activated when the AMP/ATP ratio increases [20]. In addition, AMPK is significantly activated by phosphorylation of AMPK at Thr172 which is mediated by upstream kinases [21]. The activated AMPK promotes autophagy by activating ULK1 [22]. Under stress, the activated AMPK inhibits mTOR to relieve the phosphorylation of ULK1 at Ser757, leading to the interaction of AMPK-ULK1. AMPK then activates ULK1, and eventually leads to the induction of autophagy [18]. Once activated, ULK1 enhances autophagy by activating Beclin 1-PI3KC3 complex, which is a pivotal autophagy initiating complex [23]. During autophagy, the Beclin 1-PI3KC3 complex converts LC3-I to LC3-II through lipidation [24]. ULK1 deficiency is known to block LC3 lipidation [18].

Kim et al. have reported that AMPK and mTOR are able to oppositely regulate autophagy via direct phosphorylation of ULK1 in response to nutrient signaling [18]. In addition, recent studies have shown that AMPK-mTOR-ULK1 signaling pathway initiates autophagy, which in turn exerts cardioprotection during myocardial ischemia phase [25]. In the present study, we hypothesized that LEP promotes autophagy through the activation of AMPK-mTOR-ULK1 pathways, and that activated autophagy was partially alleviated EE-induced myocardial ischemic-hypoxic injury. In addition, wortmannin was used to deepen our insights on the role of protective effects in LEP-induced myocardial protection.

## 2. Materials and Methods

### 2.1. Animals and Ethics Statement

Eight-week-old male Sprague-Dawley rats (*n* = 120), weighing 252 ± 11 g, were obtained from Shanghai Sippr-BK Laboratory Animal Co. Ltd., China. All the animals were housed in an environment with temperature of 22 ± 1°C, relative humidity of 50 ± 1% and a light/dark cycle of 12/12 hour. The rats were given standard rodent feed *ad libitum*. All animal studies were done in compliance with the ethic standard issued by following the Guide for the Care and Use of Laboratory Animals, and approved by Ethics Committee for scientific research of Shanghai University of Sport.

### 2.2. Experimental Protocol

Rats were habituated to a treadmill (15 m/minute, 10–20 minutes/day, 0% grade) for 8 consecutive days before the experiment, and then rested for 24 hours. Animals were randomly divided into the following 6 groups (*n* = 20). (i) The control (C) group: in which the rats were placed on the treadmill (without running) in order to adapt to the environment. (ii) The EE group: the rats ran at speed of 25 ~ 35 m/minute to exhaustion. The exhaustion standard was that the rats had limp limbs and thus were unable to complete the righting reflex. (iii) The LEP group: in which the rats ran at speed of 28 ~ 30 m/minute, at approximately 75% VO_2max_ intermittently for 4 repeated periods of 10 minutes (including 10 minutes rest between each period) and then had a rest for 24 hours. (iv) The LEP plus EE (LEP+EE) group: in which the exercise was performed the same way as for the LEP group, after which the rats ran to exhaustion after a 24-hour rest. (v) The wortmannin plus LEP (W+LEP) group: in which the rats were injected with wortmannin (0.7 mg/kg·w, Cell Signaling Technology) 0.5 hour before the exercise [9], which was performed the same way as for the LEP group. (vi) The wortmannin plus LEP+EE (W+LEP+EE) group: in which the rats were injected with wortmannin 0.5 hour before the exercise, which was performed the same way as for the LEP+EE group did.

The rats were anesthetized with intraperitoneal injection of 10% trichloroacetaldehyde monohydrate (400 mg/kg BW). The rats in the EE, LEP+EE, and W+LEP+EE groups were euthanized 0.5 hour after the exercise, while the rats in the LEP, W+LEP groups were euthanized 24 hours after the exercise. Briefly, approximately 5 ml blood was collected from the inferior vena cava. Ten rats from each group were selected and the rats' hearts were collected and kept at −80°C for western blot analysis. Additional ten rats from each group were subjected to perfusion fixation for histological analysis. After the rats underwent the perfusion of the 0.85% normal saline, the postcava was sheared off. When the outflow was without blood, the perfusion of 4% paraformaldehyde was performed. The heart was collected after perfusion and then was fixed in 4% paraformaldehyde for 24 hours.

### 2.3. Detection of cTnI

Plasma was centrifuged from the blood sample. The cTnI levels were analyzed by chemiluminescent immunoassay. Access AccuTnI+3 troponin I assay on an access 2 immunoassay system (Beckman Coulter, USA) was used. This assay is based on a single-step sandwich principle, i.e., paramagnetic particles were coated as the solid phase and two monoclonal cTnI antibodies. The sensitivity linear range was 0.02 ~ 100 ng/ml.

### 2.4. Hematoxylin-Basic Fuchsin-Picric Acid (HBFP) Staining and Image Analysis

The hearts were post-fixed, embedded, sectioned, dewaxed, and rehydrated. The nuclei were stained with hematoxylin. The slices were stained in 0.1% basic fuchsin and then washed in acetone, differentiated in 0.1% picric acid for 15 seconds, and washed in acetone once again. Each section was observed under a microscope (Olympus, Tokyo, Japan). For image analysis, five HBFP staining slices were randomly selected from each group and five views were selected from each slice. Image-Pro Plus software (Media Cybernetics, Silver Spring, MD, US) was used to analyze images.

### 2.5. Transmission Electron Microscopy (TEM)

Heart tissue samples (1 mm^3^) were collected from the left ventricle. The samples were fixed with glutaraldehyde phosphate buffered saline, 1% osmium tetroxide. After osmium fixation, the samples were dehydrated, resin embedded, polymerized, sectioned, and stained with uranyl acetate and lead citrate. The tissues were viewed under a TEM (H-7650, Hitachi, Tokyo, Japan).

### 2.6. Western Blot

The following antibodies were used: LKB1 (#3047), AMPK*α* (#2603), AMPK*α*^Thr172^ (#2535), mTOR (#2983), ULK1 (#8054), ULK1^Ser757^ (#14202), PI3Kinase Class III (#3358), and *β*-actin (#4970) (Cell Signaling Technology, USA). Beclin 1 and Bcl-2 (Santa Cruz Biotechnology, USA). LC3 (Novus Biologicals, USA), HRP tagged secondary antibody (Cell Signaling Technology, USA).

The myocardial tissues were homogenized in a buffer containing radioimmunoprecipitation assay cell lysate and the protease inhibitor phenylmethanesulfonyl fluoride. After homogenizing and centrifuging, the supernatant liquor was collected. The protein concentration was detected by bicinchonininc acid assay. The samples were heated at 100°C for 10 minutes. The proteins were separated using SDS-PAGE with 15% or 10%. Consequently, the protein bands were transferred to PVDF membranes. At room temperature, the membranes were blocked with 5% BSA solution for 1.5 hours and then incubated with the corresponding primary antibody (1 : 1000 dilution) at 4°C overnight. After the membranes were washed 5 times for 5 minutes with TBST, membranes were incubated with HRP-labeled secondary antibody (1 : 5000 dilution) at room temperature for 1 hour. The images were visualized and quantified by Tanon-5200 chemiluminescence imaging system (Tanon-5200 Multi, China).

### 2.7. Statistical Analysis

The SPSS19.0 statistical software (IBM, Armonk, NY, USA) was applied to the data analysis. The results were expressed as the mean ± SD. The data were compared between groups using the independent samples *T*-test. *P* < 0.05 was considered to be statistically significant.

## 3. Results

### 3.1. Relationship between Late Exercise Preconditioning-Induced Myocardial Protection and Autophagy

Plasma cTnI levels were applied to evaluate to the degree of myocardial injury induced by exercise (Table 1). Compared with the C group, the plasma cTnI levels in the EE group were remarkably higher, while no significant changes were observed in the LEP and W+LEP groups. In addition, the plasma cTnI levels in the LEP+EE group were considerably lower than in the EE group. Moreover, the results showed that inhibited autophagy led to higher cTnI levels in the W+LEP+EE group compared to the LEP+EE group.

**Figure 1:**
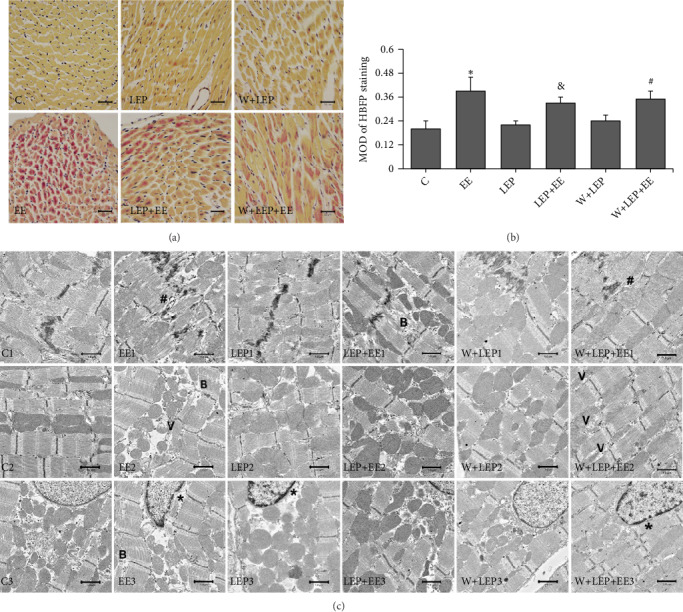
LEP initiated myocardial protection alleviated exhaustive exercise-induced myocardial injury and was partially attenuated by autophagy inhibitor. (a) The changes of myocardial ischemia-hypoxia detected by HBFP staining (×400), bar = 20 *μ*m. Nonischemic myocardial cell showed the light brown color. The ischemic tissue stained a bright crimson red color. The C, LEP, and W+LEP groups displayed a light brown color. Ischemic cardiomyocytes in the EE group stained crimson red. The LEP+EE and W+LEP+EE groups showed red patchy stain. (b) Quantitative analysis of HBFP staining. The EE group compared with the C group, the LEP+EE group compared with the EE group, and the W+LEP+EE group compared with the LEP+EE group were remarkably different. (c) The myocardial ultrastructural changes in six different conditions were observed by TEM (×3,000), bar = 1.0 *μ*m. Exhaustive exercise led to myofibrils breaks (symbol “B”), wide interfibrillar space (symbol “#”), and vacuolate mitochondria (symbol “V”). LEP had normal ultrastructure, but the heterochromatin was slightly marginalized (symbol “∗”). LEP attenuated exhaustive exercise-induced injury in the LEP + EE group. However, wortmannin alleviated the protective effect of LEP. ^∗^*P* < 0.05 vs. C, ^&^*P* < 0.05 vs. EE, ^#^*P* < 0.05 vs. LEP+EE.

HBFP staining was used to examine the changes of ischemia–hypoxia. In rat myocardium, the light brown color indicated non ischemia–hypoxia and the bright crimson color suggested the myocardial ischemia–hypoxia. Mean optical density (MOD) value was used to show the extent of ischemia–hypoxia per unit area in rat myocardium. The C group showed the light brown color (Figures 1(a)–1(c)). A few red spot stains were observed in the LEP and W+LEP groups (Figure 1(a)-LEP and W+LEP), and the MOD values in LEP and W+LEP groups remained intact compared with C group (Figure 1(b)). Yet, in the EE group, a vivid crimson red was observed in the majority of tissue (Figure 1(a)-EE). MOD values were significantly increased, indicated that the extent of ischemia–hypoxia was aggravated during exhaustive exercise (Figure 1(b)). Pretreatment with LEP before exhaustive exercise, the extent of red regions from cardiomyocytes were reduced (Figure 1(a)-LEP+EE), and the extent of ischemia–hypoxia were significantly decreased in the LEP+EE group (Figure 1(b)). In contrast, compared with the LEP+EE group, the W+LEP+EE group showed the red patchy stain (Figure 1(a)-W+LEP+EE), and the extent of ischemia–hypoxia was aggravated (Figure 1(b)). In summary, LEP-induced myocardial protection could suppress exhaustive exercise-induced ischemia–hypoxia changes. However, pretreatment with wortmannin reduced the LEP-induced beneficial effect.

Furthermore, in the C group, the ultrastructure of the cardiomyocytes had normal morphology, i.e., stable intercalated disc, regularly aligned myofibrils, normal shape of mitochondria, and well-distributed chromatin (Figure 1(c)-C1-3). Alterations in the morphology were observed in the EE group; some parts of the myofibrils were severely broken (symbol “B”) and the junction area of cells enlarged (symbol “#”); many mitochondria vacuoles appeared (symbol “V”), heterochromatin was slightly marginalized, and the nucleus was depressed. However, no nuclear pyknosis or disintegration were observed in the EE group (Figure 1(c)-EE1-3). The morphology of LEP group was normal, but the heterochromatin was slightly marginalized (symbol “∗”) (Figure 1(c)-LEP1-3). Smaller mitochondria and breakage of myofibrils caused by exhaustive exercise were preserved after LEP pretreatment in the LEP+EE group. Yet, a few myofibril breakages were still observed in this group (Figure 1(c)-LEP+EE1-3). After injecting wortmannin, no obvious ultrastructure changes were observed in the W+LEP group (Figure 1(c)-W+LEP1-3), but after the exhaustive exercise, the W+LEP+EE group showed aggravated vacuolization of mitochondria (Figure 1(c)-W+LEP+EE1-3). Collectively, these data indicated that LEP promotes autophagy and is involved in myocardial protection.

### 3.2. Changes of AMPK, mTOR and ULK1 Expression during Late Exercise Preconditioning

**Figure 2:**
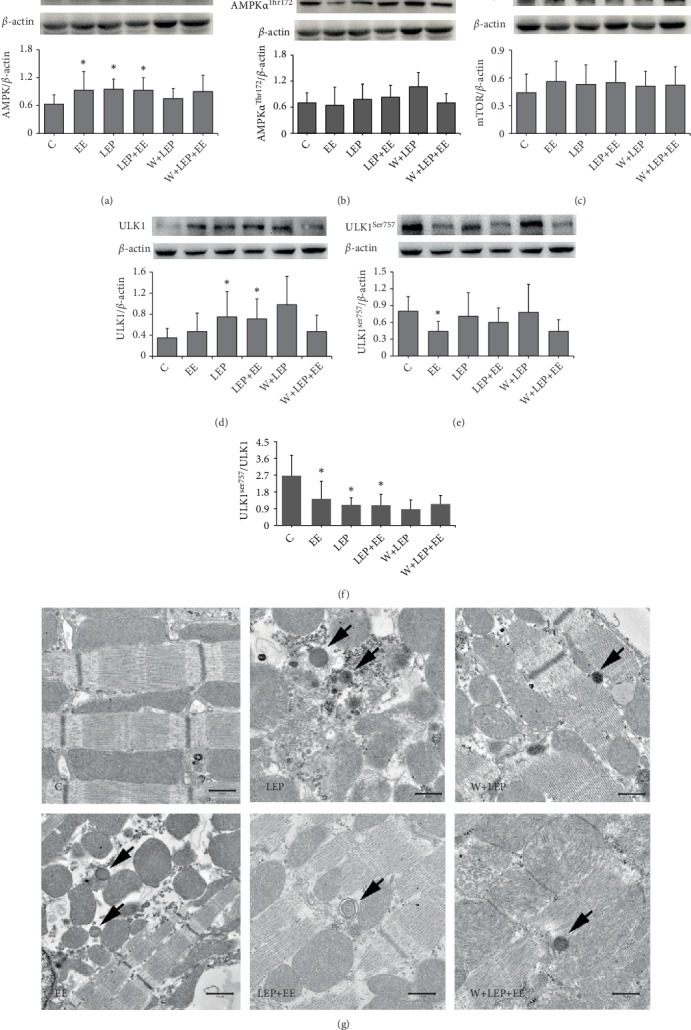
Changes of AMPK, mTOR, and ULK1 expression during late exercise preconditioning. (a–e) Images and quantitative analysis of AMPK, AMPK*α*^Thr172^, mTOR, ULK1, and ULK1^Ser757^ in rat myocardium by western blot assay. (f) Quantitative analysis of ULK1^Ser757^/ULK1. ^∗^*P* < 0.05 vs. C. (g) Autophagosomes and lysosomes were observed from these groups by TEM. (×5,000), bar = 1.0 *μ*m.

When cellular energy is depleted, AMPK is activated and it then inhibits mTOR, therefore, reducing the phosphorylation of ULK1 at Ser757, AMPK then activates ULK1. To explore whether LEP promoted autophagy through the AMPK-mTOR-ULK1 pathway, AMPK, AMPK*α*^Thr172^, mTOR, ULK1, and ULK1^Ser757^ protein expression were examined using western blot (Figures 2(a)–2(e)). AMPK and mTOR play important roles in regulation of autophagy.  Figure 2 showed that the expressions of AMPK in the EE, LEP, and LEP+EE groups were significantly higher than in the C group (Figure 2(a)); however, no significant change in the AMPK*α*^Thr172^ levels was found among groups (Figure 2(b)). Although the mTOR levels remained unchanged among groups (Figure 2(c)), the ULK1^ser757^/ULK1 ratio in the EE, LEP, and LEP+EE groups was significantly lower than in the C group (Figure 2(f)). Thus, the inhibition of mTOR on ULK1 was attenuated after exercise. Moreover, the EE group showed a significant decrease in ULK1^Ser757^ levels compared to the C group (Figure 2(e)). In addition, ULK1 expression in the LEP and LEP+EE groups were significantly higher compared to the C group (Figure 2(d)). Collectively, these results suggest that AMPK-mTOR-ULK1 pathway is activated during LEP, EE, and LEP+EE.

Furthermore, almost no autophagosomes and lysosomes were observed by TEM in the C group. Yet, autophagosomes (as indicated by black arrows) were observed in LEP+EE group; lysosomes (as indicated by black arrows) were observed in other groups (Figure 2(g)). In summary, these results implied that the regulation of LEP on autophagy occurred through the AMPK-mTOR-ULK1 pathway.

### 3.3. Changes of Associated Proteins Expression during Late Exercise Preconditioning-Induced Myocardial Protection

**Figure 3:**
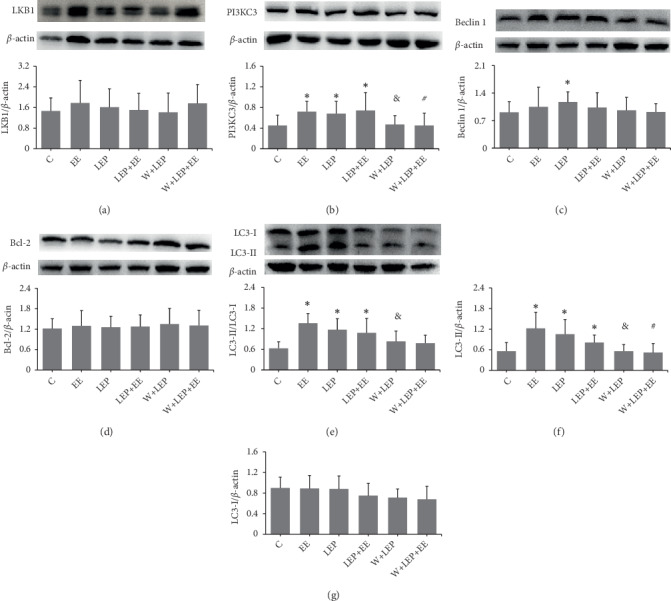
Changes of associated proteins expression during late exercise preconditioning-induced myocardial protection. (a-d and f-g) Images and quantitative analysis of LKB1, PI3KC3, Beclin 1, Bcl-2LC3-II, and LC3-I by western blot assay. (e) Quantitative analysis of LC3-II/LC3-I. ^∗^*P* < 0.05 vs. C, ^&^*P* < 0.05 vs. EE, ^#^*P* < 0.05 vs. LEP+EE.

In order to investigate the role of associated proteins in regulating autophagy, we detected LKB1, Beclin 1, Bcl-2, PI3KC3, and LC3 in myocardium using western blot. The protein expression is shown in Figure 3. No significant changes in LKB1 and Bcl-2 levels were detected in any of the groups (Figures 3(a) and 3(c)). The LEP group showed an increase in Beclin 1 levels compared to the C group (*P* < 0.05) (Figure 3(b)). PI3KC3 levels in LEP, EE, and LEP+EE groups were remarkably higher compared to the C group (Figure 3(d)). LC3-II was often used as the indicator of autophagosomes [26]. The LC3-II levels and LC3-II/LC3-I ratio in the LEP group were higher than in the C group (Figures 3(e) and 3(f)), which implied that LEP increased autophagosomes and promoted autophagic activity. The similar results were found in the EE and LEP+EE groups (Figures 3(e) and 3(f)). Nevertheless, under the influence of wortmannin, PI3KC3 expression in the W+LEP and W+LEP+EE groups were lower compared to the LEP and LEP+EE groups, respectively (Figure 3(d)). In addition, the LC3-II levels and LC3-II/LC3-I ratio induced by LEP were abolished by wortmannin in the W+LEP group (Figures 3(e) and 3(f)). Although the LC3-II/LC3-I ratio showed no remarkable change, the decrease in LC3-II levels resulted from the autophagy inhibitor wortmannin in the W+LEP+EE group (Figures 2(e) and 2(f)). These results indicated that pretreatment with wortmannin inhibited autophagy during W+LEP and W+LEP+EE. There was no considerable changes in the LC3-I expression among groups (Figure 2(g)).

## 4. Discussion

### 4.1. Late Exercise Preconditioning Alleviates Exhaustive Exercise-Induced Myocardial Ischemic-Hypoxic Injury

Preclinical evidence indicates that exercise per week can confer immediate strong cardioprotection [1]. However, acute bouts of excessive exhaustive exercise may cause myocardial injury [27]. cTnI, also known as cardiac biomarker, can quickly enter the blood upon cardiac injury [28] and thus is commonly employed as indicator of the exercised-induced myocardial injury [29]. HBFP staining can be used for detection of the early stages of myocardial ischemia. In this study, cTnI, HBFP staining, and TEM were used for comprehensive evaluation of myocardial ischemic-hypoxic injury. We found severe ultrastructure damages after exhaustive exercise, particularly breakage of myocardial fibers [2], which led to cTnI release from cardiomyocytes into the circulation [9]. Furthermore, HBFP staining and MOD value showed that exhaustive exercise caused severe myocardial ischemia-hypoxic changes. Collectively, these data indicated that exhaustive exercise might induce acute myocardial ischemic-hypoxic injury. However, no pyknosis or disintegration of the nucleus were observed in the EE group, suggesting that exhaustive exercise-induced myocardial ischemia-hypoxic injury was reversible.

Emerging evidence shows that exercise preconditioning can create clinically relevant cardioprotection, such as alleviation of ischemic myocardial injury in rat [30], reduction of the infarct size by 76% and 52% in the early and late cardiac preconditioning, respectively [6]. In this study, no changes in specific injury phenotypes, such as cTnI levels, ischemia–hypoxia and myofibril breakage were observed after 24 hours of EP, thus suggesting that LEP was safe to the myocardium. However, LEP led to mild changes of myocardial ultrastructure. In the LEP+EE group, pretreatment with LEP before exhaustive exercise obviously suppressed myocardial ischemic-hypoxic injury and decreased the release of cTnI, as well as reduced ultrastructure changes.

Under some conditions, such as acute ischemia, long or short-term exercise, autophagy can be activated and in turn can cause myocardial protection [31–33]. Therefore, LEP-induced myocardial protection is presumably associated with autophagy. In this study, two additional groups of rats, namely the W+LEP group and W+LEP+EE group (LEP and LEP+EE models were treated with wortmannin, respectively) were used. Wortmannin has been reported to reduce the myocardial protective effects of ischemic conditioning by inhibiting autophagy [34]. In this study, the LC3-II levels were significantly decreased in the W+LEP and W+LEP+EE groups, thus suggesting that pretreatment with wortmannin might inhibit LEP- and LEP+EE-induced formation of autophagosomes. Combined with the previously mentioned injuries in the W+LEP+EE group, these results suggested that LEP-induced autophagy was partially involved in myocardial protection, while the beneficial effects were suppressed by the application of autophagy inhibitor.

### 4.2. Late Exercise Preconditioning Promotes Autophagy through the Activation of the AMPK-mTOR-ULK1 Pathway

In the present study, we demonstrated that LEP activated autophagy through activation of the AMPK-mTOR-ULK1 pathway in rat myocardium. Some studies have reported that AMPK is activated in mouse cardiomyocytes during ischemia, meanwhile autophagy is upregulated [35]. During I/R, autophagy is induced via activation of AMPK and inhibition of mTOR [36], which is why the inhibition of mTOR by AMPK during LEP may promote autophagy.

LKB1, one of major upstream kinases for AMPK, can strengthen AMPK activation by phosphorylation of Thr172 on AMPK [37]. In this study, we found that LEP may increase the AMPK levels, but it has no effect on LKB1 and AMPK*α*^Thr172^. Some previous studies have suggested that activation of AMPK may be regulated by the increase of AMP/ATP ratio [35, 38, 39]. Thus, in this study, AMPK activation was independent of phosphorylation of AMPK at Thr172 by LKB1 during LEP. In the LEP group, no changes in mTOR levels were observed [40]; however, the ULK1^Ser757^/ULK1 ratio was remarkably decreased. These results suggested that AMPK inhibits the activity of mTOR during LEP [41], which then alleviates the inhibition of ULK1 phosphorylation. Collectively, LEP upregulates ULK1 activity by increasing AMPK activity, and inhibits mTOR-dependent phosphorylation of ULK1 at Ser757 [17, 41]. In addition, LEP also increased ULK1 expression. Taken together, our findings suggested that LEP activated the AMPK-mTOR-ULK1 pathway. These effects of LEP may involve the increase of AMPK and ULK1 expression, no changes in mTOR levels, as well as decrease of phosphorylation of ULK1 at Ser757. The activation of the AMPK-mTOR-ULK1 pathway was involved in the regulation of autophagy during LEP, as evidenced by the increase of LC3-II/LC3-I ratio and LC3-II levels. Similar results were also observed in the EE and LEP+EE groups. Thus, EE and LEP+EE could promote autophagy by activating the AMPK-mTOR-ULK1 pathway. In addition, autophagosomes was observed during LEP+EE, further suggesting that LEP pretreatment before exhaustive exercise promoted the formation of autophagosomes.

### 4.3. Autophagy Induced by Late Exercise Preconditioning Participates in Myocardial Protection

In this study, elevated autophagy partially participated in LEP-induced myocardial protection against exhaustive exercise-induced myocardial injury. In the heart, AMPK- and mTOR-dependent autophagy are part of the cardioprotective effects during ischemia [3, 42]. Previous research has shown that autophagy, initiated by the AMPK-mTOR-ULK1 signaling pathway during myocardial ischemia phase has a protective effect [25]. In the present study, LEP enhanced autophagy through the AMPK-mTOR-ULK1 pathway, but whether autophagy had a beneficial role during subsequent exhaustive exercise needs to be further discussed. Accordingly, in this study, we used wortmannin, a classical autophagy inhibitor to investigate the contribution of autophagy to LEP-induced myocardial protection.

The ULK1 complex can translate upstream signals from AMPK and mTORC1 into autophagosome formation [17]. Once activated, ULK1 enhances the formation of autophagosome by activating Beclin 1-PI3KC3 complex [23]. Under normal conditions, Beclin 1 is inhibited by Bcl-2, which then downregulates the autophagy by inhibiting the interaction between Beclin 1 and PI3KC3 [43, 44]. However, during repeated I/R, Beclin 1 in the heart of rat is markedly increased, thus enhancing autophagy [45]. In this study, the increase of Beclin 1 and PI3KC3 levels could form the new Beclin l-PI3KC3 complex during LEP, which then promoted LC3-I to LC3-II [23]. Accordingly, enhanced autophagy during LEP may be involved in the endogenic stimulation of subsequent exhaustive exercise. Nevertheless, no alterations in Beclin 1 levels were found in the LEP+EE group, indicating that Beclin 1 interacted with PI3KC3 to form the new complex during LEP, thereby the changes were not observable in the studied time phase. Combined with the previously mentioned injury phenotypes in the LEP+EE group, our results indicated that LEP-promoted autophagy through the AMPK-mTOR-ULK1 pathway was partially alleviated exhaustive exercise-induced myocardial ischemic-hypoxic injury.

After pretreatment with wortmannin, PI3KC3 activity, and LC3 lipidation were blocked during W+LEP and W+LEP+EE [9]. Wortmannin significantly inhibited the conversion of LC3-I to LC3-II, induced by LEP and LEP+EE, thereby indicating autophagic obstruction during W+LEP and W+LEP+EE. Finally, combined with the previously mentioned injury in the W+LEP+EE group, inhibition of autophagy by wortmannin attenuated LEP-induced myocardial protection. Taken together, LEP promoted autophagy through the activation of AMPK-mTOR-ULK1 pathway, and that activated autophagy was partially involved in the myocardial protection against exhaustive exercise-induced myocardial injury.

In addition, previous study has reported that the efficiency of autophagosome clearance is decreased during reperfusion, which contributes to cardiomyocyte death [46]. In this study, Beclin 1, an autophagic inducer, was not altered during exhaustive exercise [47], but the LC3-II levels and LC3-II/LC3-I ratio were increased. This implied that LC3 accumulation might likely occur from a block in autophagosome degradation during exhaustive exercise [9, 36, 46], which in turn increased proteotoxicity of misfolded/damaged proteins and aggravated myocardial injury. Yet, the details of the mechanism on the up-regulation of autophagy levels during exhaustive exercise need to be further investigated.

## 5. Conclusions

This study demonstrated that LEP-induced myocardial protection exerts protective effects through the reduction of exhaustive exercise-induced myocardial ischemic-hypoxic injury. LEP promotes autophagy through the activation of AMPK-mTOR-ULK1 pathway, and that activated autophagy is partially involved in myocardial protection. LEP offers its beneficial effects involve the increase of AMPK activity and decrease of mTOR-dependent phosphorylation of ULK1 at Ser757, as well as an enhancement of AMPK and ULK1 expression.

## Figures and Tables

**Table 1:** Changes in plasma cTnI levels of each group.

Group	*n*	cTnI (ng/ml)
C	20	0.006 ± 0.006
EE	18	19.207 ± 13.753^∗^
LEP	20	0.012 ± 0.017
LEP+EE	19	2.270 ± 1.384^&^
W+LEP	20	0.028 ± 0.032
W+LEP+EE	17	5.645 ± 2.962^#^

^∗^ *P* < 0.05 vs. C, ^&^*P* < 0.05 vs. EE, ^#^*P* < 0.05 vs. LEP+EE.

## Data Availability

The data used to support the findings of this study are available from the corresponding author upon request.

## References

[1] 
Thijssen D. H. J., Redington A., George K. P., Hopman M. T. E., Jones H. (2018). Association of exercise preconditioning with immediate cardioprotection: a review. *JAMA Cardiology*.

[2] Hao Z., Pan S.-S., Shen Y.-J., Ge J. (2014). Exercise preconditioning-induced early and late phase of cardioprotection is associated with protein kinase C epsilon translocation. *Circulation Journal*.

[3] Yuan Y., Pan S. S., Shen Y. J. (2018). Cardioprotection of exercise preconditioning involving heat shock protein 70 and concurrent autophagy: a potential chaperone-assisted selective macroautophagy effect. * The Journal of Physiological Sciences*.

[4] Domenech R., Macho P., Schwarze H., Sánchez G. (2002). Exercise induces early and late myocardial preconditioning in dogs. * Cardiovascular Research*.

[5] Marongiu E., Crisafulli A. (2014). Cardioprotection acquired through exercise: the role of ischemic preconditioning. *Current Cardiology Reviews*.

[6] Parra V. M., Macho P., Sánchez G., Donoso P., Domenech R. J. (2015). Exercise preconditioning of myocardial infarct size in dogs is triggered by calcium. * Journal of Cardiovascular Pharmacology*.

[7] McGinnis G. R., Ballmann C., Peters B. (2015). Interleukin-6 mediates exercise preconditioning against myocardial ischemia reperfusion injury. * American Journal of Physiology-Heart and Circulatory Physiology*.

[8] Parra V. M., Macho P., Domenech R. J. (2010). Late cardiac preconditioning by exercise in dogs is mediated by mitochondrial potassium channels. * Journal of Cardiovascular Pharmacology*.

[9] Yuan Y., Pan S. S. (2018). Parkin mediates mitophagy to participate in cardioprotection induced by late exercise preconditioning but Bnip3 does not. * Journal of Cardiovascular Pharmacology*.

[10] Klionsky D. J. (2007). Autophagy: from phenomenology to molecular understanding in less than a decade. * Nature Reviews Molecular Cell Biology*.

[11] Campos J. C., Queliconi B. B., Bozi L. H. M. (2017). Exercise reestablishes autophagic flux and mitochondrial quality control in heart failure. *Autophagy*.

[12] Hamacher-Brady A., Brady N. R., Gottlieb R. A. (2006). Enhancing macroautophagy protects against ischemia/reperfusion injury in cardiac myocytes. * Journal of Biological Chemistry*.

[13] Godar R. J., Ma X., Liu H. (2015). Repetitive stimulation of autophagy-lysosome machinery by intermittent fasting preconditions the myocardium to ischemia-reperfusion injury. *Autophagy*.

[14] Yan L., Sadoshima J., Vatner D. E., Vatner S. F. (2009). Autophagy in ischemic preconditioning and hibernating myocardium. *Autophagy*.

[15] Park J. M., Jung C. H., Seo M. (2016). The ULK1 complex mediates MTORC1 signaling to the autophagy initiation machinery via binding and phosphorylating ATG14. * Autophagy*.

[16] Zachari M., Ganley I. G. (2017). The mammalian ULK1 complex and autophagy initiation. * Essays In Biochemistry*.

[17] Egan D., Kim J., Shaw R. J., Guan K.-L. (2011). The autophagy initiating kinase ULK1 is regulated via opposing phosphorylation by AMPK and mTOR. * Autophagy*.

[18] Kim J., Kundu M., Viollet B., Guan K.-L. (2011). AMPK and mTOR regulate autophagy through direct phosphorylation of Ulk1. * Nature Cell Biology*.

[19] Rocchi A., He C. (2017). Regulation of exercise-induced Autophagy in skeletal muscle. * Current Pathobiology Reports*.

[20] Moller A. B., Vendelbo M. H., Christensen B. (2015). Physical exercise increases autophagic signaling through ULK1 in human skeletal muscle. * Journal of Applied Physiology*.

[21] Wang S., Song P., Zou M. H. (2012). AMP-activated protein kinase, stress responses and cardiovascular diseases. * Clinical Science*.

[22] Shang L., Wang X. (2011). AMPK and mTOR coordinate the regulation of Ulk1 and mammalian autophagy initiation. * Autophagy*.

[23] Hurley J. H., Young L. N. (2017). Mechanisms of autophagy initiation. * Annual Review of Biochemistry*.

[24] Boya P., Reggiori F., Codogno P. (2013). Emerging regulation and functions of autophagy. * Nature Cell Biology*.

[25] Ma S., Wang Y., Chen Y., Cao F. (2015). The role of the autophagy in myocardial ischemia/reperfusion injury. *Biochimica et Biophysica Acta (BBA)–Molecular Basis of Disease*.

[26] Wang J. F., Mei Z.-G., Fu Y. (2018). Puerarin protects rat brain against ischemia/reperfusion injury by suppressing autophagy via the AMPK-mTOR-ULK1 signaling pathway. * Neural Regeneration Research*.

[27] Whyte G. P. (2008). Clinical significance of cardiac damage and changes in function after exercise. *Medicine & Science in Sports & Exercise*.

[28] Li T., Zhu D., Zhou R., Wu W., Li Q., Liu J. (2012). HBOC attenuates intense exercise-induced cardiac dysfunction. * International Journal of Sports Medicine*.

[29] Shave R., Baggish A., George K. (2010). Exercise-induced cardiac troponin elevation: evidence, mechanisms, and implications. * Journal of the American College of Cardiology*.

[30] Yamashita N., Hoshida S., Otsu K., Asahi M., Kuzuya T., Hori M. (1999). Exercise provides direct biphasic cardioprotection via manganese superoxide dismutase activation. * The Journal of Experimental Medicine*.

[31] Sciarretta S., Hariharan N., Monden Y., Zablocki D., Sadoshima J. (2011). Is autophagy in response to ischemia and reperfusion protective or detrimental for the heart?. *Pediatric Cardiology*.

[32] Chen C. Y., Hsu H.-C., Lee B.-C. (2010). Exercise training improves cardiac function in infarcted rabbits: involvement of autophagic function and fatty acid utilization. * European Journal of Heart Failure*.

[33] Lee Y., Kang E.-B., Kwon I., Cosio-lima L., Cavnar P., Javan G. T. (2016). Cardiac kinetophagy coincides with activation of anabolic signaling. * Medicine & Science in Sports & Exercise*.

[34] Velez D. E., Hermann R., Frank M. B. (2016). Effects of wortmannin on cardioprotection exerted by ischemic preconditioning in rat hearts subjected to ischemia-reperfusion. * Journal of Physiology and Biochemistry*.

[35] Matsui Y., Takagi H., Qu X. (2007). Distinct roles of autophagy in the heart during ischemia and reperfusion: roles of AMP-activated protein kinase and Beclin 1 in mediating autophagy. * Circulation Research*.

[36] Hao M., Zhu S., Hu L., Zhu H., Wu X., Li Q. (2017). Myocardial ischemic postconditioning promotes autophagy against ischemia reperfusion injury via the activation of the nNOS/AMPK/mTOR pathway. * International Journal of Molecular Sciences*.

[37] Carling D., Sanders M. J., Woods A. (2008). The regulation of AMP-activated protein kinase by upstream kinases. * International Journal of Obesity*.

[38] Ma X., Fu Y., Xiao H. (2015). Cardiac fibrosis alleviated by exercise training is AMPK-dependent. *PLoS One*.

[39] Cui Y. C., Pan C.-S., Yan L. (2017). Ginsenoside Rb1 protects against ischemia/reperfusion-induced myocardial injury via energy metabolism regulation mediated by RhoA signaling pathway. *Scientific Reports*.

[40] Liao J., Li Y., Zeng F., Wu Y. (2015). Regulation of mTOR pathway in exercise-induced cardiac hypertrophy. * International Journal of Sports Medicine*.

[41] Yuan J., Zhao X., Hu Y. (2018). Autophagy regulates the degeneration of the auditory cortex through the AMPK-mTOR-ULK1 signaling pathway. *International Journal of Molecular Medicine*.

[42] Sciarretta S., Volpe M., Sadoshima J. (2014). Mammalian target of rapamycin signaling in cardiac physiology and disease. * Circulation Research*.

[43] Mei Y., Glover K., Su M., Sinha S. C. (2016). Conformational flexibility of BECN1: essential to its key role in autophagy and beyond. * Protein Science*.

[44] Maejima Y., Isobe M., Sadoshima J. (2016). Regulation of autophagy by Beclin 1 in the heart. * Journal of Molecular and Cellular Cardiology*.

[45] Gurusamy N., Lekli I., Gorbunov N. V., Gherghiceanu M., Popescu L. M., Das D. K. (2009). Cardioprotection by adaptation to ischaemia augments autophagy in association with BAG-1 protein. *Journal of Cellular and Molecular Medicine*.

[46] Ma X., Liu H., Foyil S. R., Godar R. J., Weinheimer C. J., Diwan A. (2012). Autophagy is impaired in cardiac ischemia-reperfusion injury. *Autophagy*.

[47] Xu Q., Li X., Lu Y. (2015). Pharmacological modulation of autophagy to protect cardiomyocytes according to the time windows of ischaemia/reperfusion. *British Journal of Pharmacology*.

